# Heat-activated nanomedicine formulation improves the anticancer potential of the HSP90 inhibitor luminespib in vitro

**DOI:** 10.1038/s41598-021-90585-w

**Published:** 2021-05-27

**Authors:** Brittany Epp-Ducharme, Michael Dunne, Linyu Fan, James C. Evans, Lubabah Ahmed, Pauric Bannigan, Christine Allen

**Affiliations:** Leslie Dan Faculty of Pharmacy, University of Toronto, Toronto, ON M5S 3M2 Canada

**Keywords:** Nanoparticles, Non-small-cell lung cancer, Nanotechnology in cancer, Drug delivery, Chemotherapy

## Abstract

The heat shock protein 90 inhibitor, luminespib, has demonstrated potent preclinical activity against numerous cancers. However, clinical translation has been impeded by dose-limiting toxicities that have necessitated dosing schedules which have reduced therapeutic efficacy. As such, luminespib is a prime candidate for reformulation using advanced drug delivery strategies that improve tumor delivery efficiency and limit off-target side effects. Specifically, thermosensitive liposomes are proposed as a drug delivery strategy capable of delivering high concentrations of drug to the tumor in combination with other chemotherapeutic molecules. Indeed, this work establishes that luminespib exhibits synergistic activity in lung cancer in combination with standard of care drugs such as cisplatin and vinorelbine. While our research team has previously developed thermosensitive liposomes containing cisplatin or vinorelbine, this work presents the first liposomal formulation of luminespib. The physico-chemical properties and heat-triggered release of the formulation were characterized. Cytotoxicity assays were used to determine the optimal drug ratios for treatment of luminespib in combination with cisplatin or vinorelbine in non-small cell lung cancer cells. The formulation and drug combination work presented in this paper offer the potential for resuscitation of the clinical prospects of a promising anticancer agent.

## Introduction

Heat shock protein 90 (HSP90) is a 90 kDa molecular chaperone responsible for the folding, stabilization, and activation of hundreds of client proteins involved in cell cycle control, signal transduction, and DNA damage repair pathways^1–5^. Many oncoproteins are client proteins of HSP90^6–8^, thereby positioning HSP90 as a central target in various cancers. Over the last 25 years, numerous HSP90 inhibitors (HSP90i) have been discovered and studied preclinically, 18 of which have entered clinical trials^9^. In the clinic, HSP90i have not been able to strike an effective balance between systemic toxicity and clinical efficacy^10,11^. Luminespib (LUM) is a second-generation HSP90i co-developed by the Institute of Cancer Research (London, United Kingdom) and Vernalis Research (subsequently licensed to Novartis). Preclinically, LUM has demonstrated antitumor activity in various tumor models^12–20^. LUM has been assessed in 27 clinical trials (Phase 1 and 2), as both a monotherapy and in combination with chemotherapy, in various cancers^21–33^. Although treatment with LUM led to partial response and stable disease, notably in patients with non-small cell lung cancer (NSCLC) with mutations in EGFR and ALK^21^, it failed to meet clinical trial endpoints, and its systemic administration resulted in a high occurrence of ophthalmological toxicities hindering further development^10,25–28^.

Nanomedicine formulation strategies are a promising approach to ameliorate the systemic toxicity associated with HSP90i while simultaneously allowing for enhanced drug accumulation at the tumor site^34^. Presently, liposomes represent the most clinically successful nanomedicine formulations including Doxil^®^ and the more recently approved Vyxeos^®^^35^. Several liposomal formulations encapsulating HSP90i have been developed^36–41^, but a liposome formulation for LUM has not yet been reported. Despite their success, conventional liposomes have inherent limitations such as a reliance on the heterogeneous enhanced permeability and retention (EPR) effect^42^ and incomplete drug release^43,44^. Thermosensitive liposomes have been developed in order to overcome these inherent weaknesses. These heat-sensitive nanoparticles are able to entrap drugs in their aqueous core below the gel to liquid crystalline transition temperature (T_m_) of the lipid bilayer. When the liposomes are heated, via an external stimulus, to temperatures above their T_m_, the drug cargo is released into the surrounding tumor vasculature from where it is able to extravasate into the tumor tissue^45^. ThermoDox^®^, a thermosensitive liposome containing doxorubicin, is the most clinically advanced thermosensitive liposome formulation, having undergone clinical trials in various solid tumors (NCT00826085, NCT00441376, NCT02181075, NCT00617981, NCT00346229, NCT00093444). While the ultimate clinical fate of ThermoDox is not yet known, this landmark thermosensitive drug carrier has left its mark on the drug development community by inspiring the development of many similar heat-activated, rapid-release liposome formulations encapsulating both chemo- and molecular therapeutics^36,46–49^.

It has been suggested that HSP90i may be best utilized in combination with chemo- or molecular therapeutics as the disruption of HSP90 client proteins may enhance the cytotoxicity of other therapies^50,51^. For example, HSP90 inhibition has been shown to lead to AKT inhibition and enhanced apoptosis in cancer cells treated with microtubule inhibitors^52,53^. Combination treatments are commonly used in cancer therapy. These include combinations of two or more chemotherapeutics or combining chemotherapeutics with other treatment modalities such as radiotherapy, hyperthermia (HT), molecular therapies or immunotherapies^54–57^. Although some combinations work together to achieve additive or synergistic effects, others may result in antagonistic effects, and therefore all combinations must be carefully selected to achieve maximum efficacy with minimal off-target toxicities^58^. Our previous work has demonstrated that thermosensitive liposomes are an efficient strategy to co-deliver chemotherapeutics and molecular therapies to tumors and that efficacy can be enhanced by HT^36^.

As lung cancer is the leading cause of cancer-related deaths^59^, current standard-of-care treatments including surgery, radiation, and chemotherapeutic regimens provide insufficient clinical efficacy and novel approaches are required^60–62^. In the preclinical setting, LUM is an effective anticancer treatment for NSCLC^63^. Additionally, some clinical activity has also been observed in NSCLC patients^28^. However, in order to improve the anticancer potential of LUM, an improved formulation and the identification of an effective combination treatment is needed. In the present study, a thermosensitive liposomal formulation containing LUM was developed to enhance the efficacy of LUM, while simultaneously mitigating off-target toxicities. In order to increase the aqueous solubility of LUM, a mesylate salt of the drug was utilized. In NSCLC cell monolayers, LUM was combined with standard of care chemotherapeutics [i.e., cisplatin (CDDP) and vinorelbine (VRL)], as well as mild HT, in order to investigate the ability of these three combinations to enhance the efficacy of LUM. As a result, a stable liposome formulation of LUM was developed that provides rapid and efficient drug release upon mild heating and specific ratios of LUM with both CDDP and VRL that result in synergistic activity were identified.

## Results

### Physicochemical characterization and stability of thermosensitive liposomal formulation of LUM

**Figure 1:**
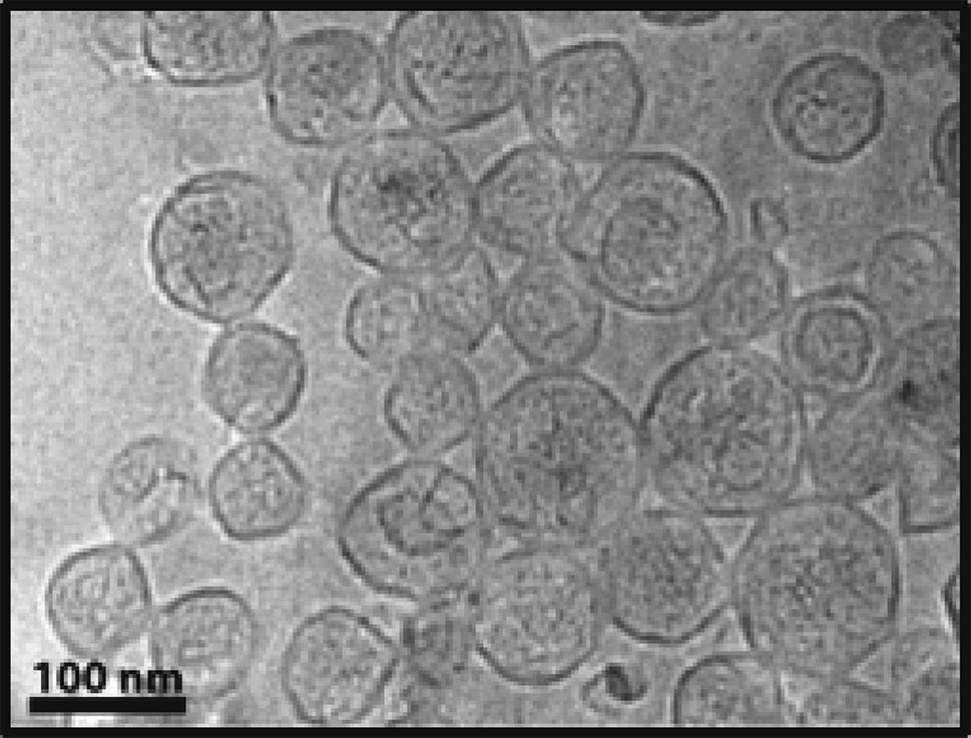
Representative cryo-TEM micrograph of the thermoLUM liposomes.

The physicochemical characteristics of the newly developed thermosensitive liposome formulation of LUM (thermoLUM) are summarized in Table 1. Thermosensitive liposomes consisting of 1,2-dipalmitoyl-sn-glycero-3-phosphocholine (DPPC), 1-stearoyl-2-hydroxy-sn-glycero-3-phosphatidylcholine (MSPC), and *N*-(carbonyl-methoxypolyethylene glycol 2000)-1,2-distearoyl-sn-glycero-3 phosphoethanolamine (mPEG_2000_-DSPE) at an 86:10:4 molar ratio were prepared with a solution of TEA_8_SOS (pH 5.7) in the internal aqueous volume and an external liposome solution of HEPES-buffered saline (HBS) solution (pH 7.4). This allowed for the active loading of LUM, which resulted in high loading efficiency and a drug-to-lipid ratio of 1:24, equivalent to approximately 5000 LUM molecules per liposome. The liposomes were found to have a negative ζ-potential of − 31 ± 2 mV, and a diameter of 102 ± 2 nm, with a narrow size distribution [polydispersity index (PDI) = 0.08 ± 0.03]. The T_m_ of the lipid bilayer was found to be in the range of mild HT (40.10 ± 0.13 °C). Cryo-TEM was conducted to confirm the morphology of the liposomes. As seen in Fig. 1, the liposomes were found to be roughly spherical in shape.

**Table 1:** Summary of the experimentally measured physiochemical properties of thermoLUM.

Parameters	Value
Lipid composition	86:10:4 mol% DPPC:MSPC:mPEG2000-DSPE
Loading efficiency	86 ± 4%*
Drug to lipid molar ratio	1 to 24.2 ± 0.6*
Liposome diameter	102 ± 2 nm*
PDI	0.08 ± 0.03*
ζ-Potential	− 31 ± 2 mV*
Tm (unloaded liposomes)	41.09 ± 0.04 °C*
Tm (thermoLUM)	40.10 ± 0.13 °C*
Drug molecules per liposome	5000 ± 200

*Error values represent the standard deviation (SD) obtained from three or more independent batches of liposomes.

**Figure 2:**
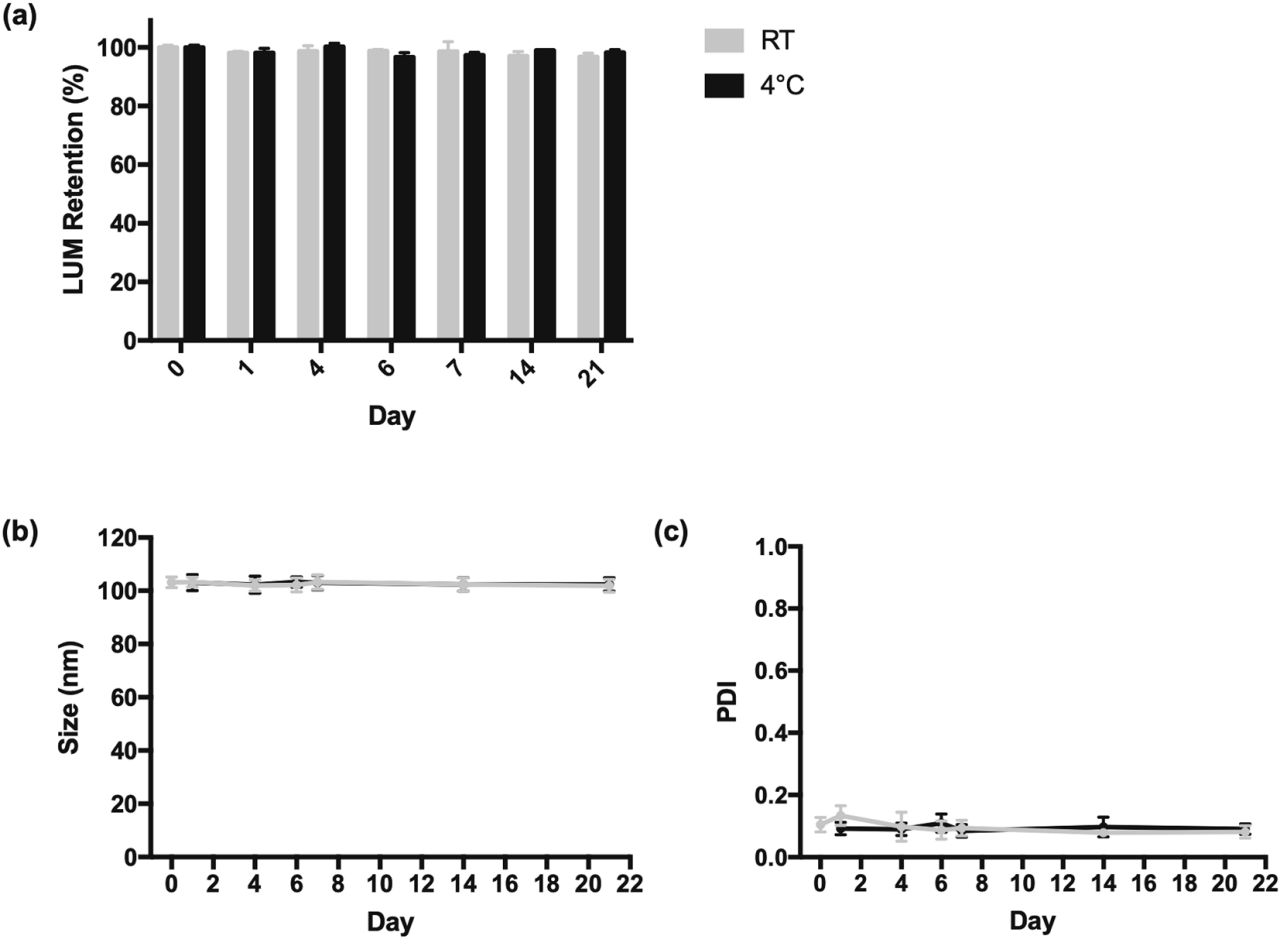
Long-term stability of thermoLUM stored at room temperature (RT, grey) or 4 °C (black) over 21 days. The liposome-encapsulated drug was separated from unencapsulated drug via size exclusion chromatography. The concentration of LUM was detected via HPLC analysis to determine the amount of drug retained in the liposomes at each timepoint (**a**). The size (**b**) and PDI (**c**) of thermoLUM were determined via DLS. The level of encapsulated drug, size, and PDI of liposomes were found to be stable over the 21-day period.

ThermoLUM was found to be stable over 21 days when stored at room temperature (RT) and 4 °C (Fig. 2). At day 21, thermoLUM liposomes were found to retain approximately 97% of encapsulated drug when stored at RT, and approximately 99% when stored at 4 °C. Minimal fluctuation in the size and PDI of thermoLUM liposomes was observed over 21 days.

### LUM release from thermosensitive liposomes at hyperthermic temperatures

**Figure 3:**
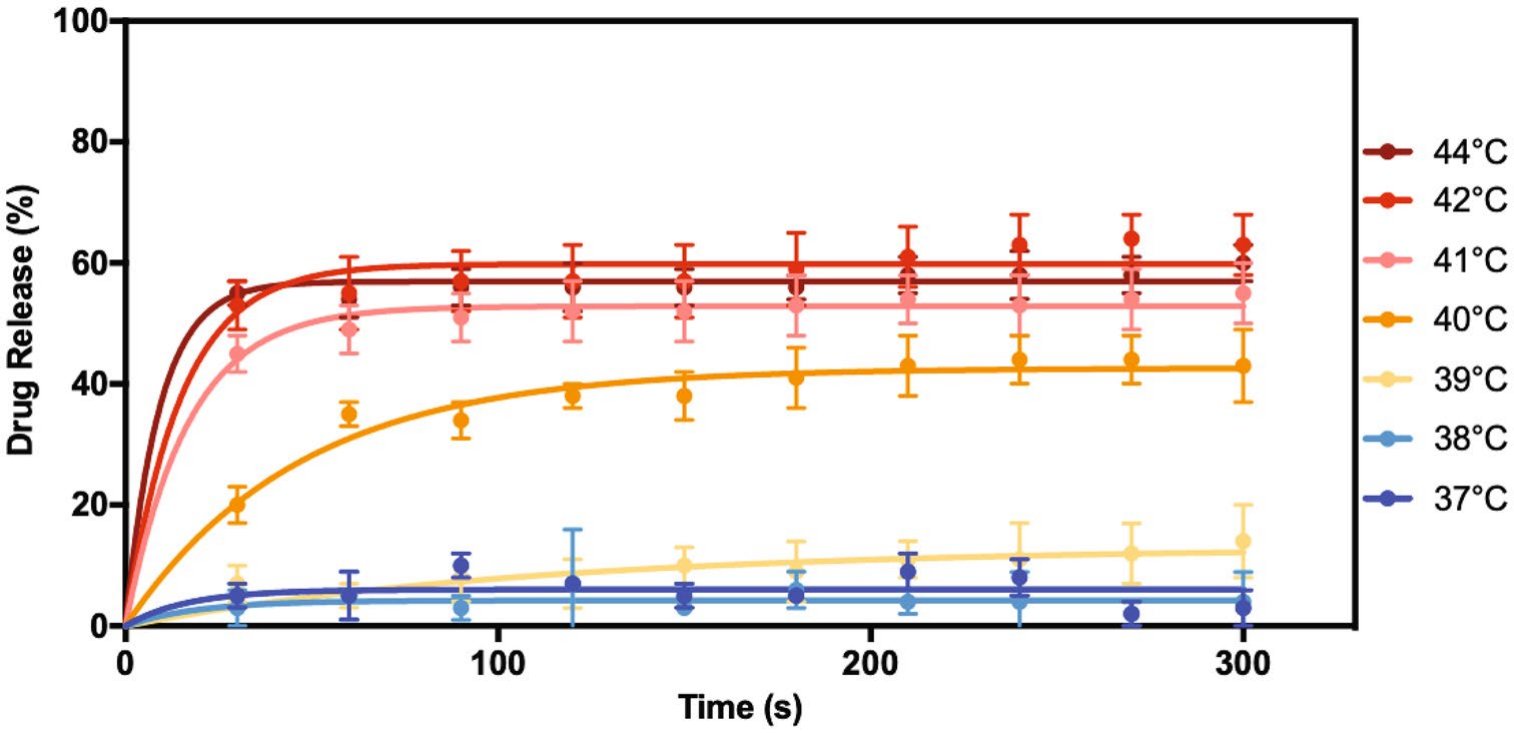
Drug release from thermoLUM incubated at 37–44 °C in 45 mg/mL BSA over 300 s. Samples were collected at 30 s intervals. The liposome-encapsulated drug was separated from unencapsulated drug via size exclusion chromatography. The concentration of LUM was detected via HPLC analysis to determine the amount of drug release from the liposomes at each timepoint. The liposomes were found to release less than 15% of drug over 5 min below 39 °C, while burst release was observed at 41–44 °C. Data shown represent the mean ± SD (n = 3).

An in vitro release study was performed to determine the stability of the thermoLUM liposomes in the presence of protein [i.e., 45 mg/mL bovine serum albumin (BSA)] at 37 °C. ThermoLUM was found to retain most of the encapsulated drug at 37 °C, with less than 15% released after 60 min (see Supplementary Fig. S1). The heat triggered drug release was assessed across a range of hyperthermic temperatures (i.e., 1 °C increments between 38 and 44 °C) for 5 min (Fig. 3). The liposomes were found to release a minimal amount of drug at 37–39 °C (i.e., less than 15% release over 5 min). At 40 °C, 20% of the drug was released in the first 30 s, and release continued, reaching a maximum of 44% at 4 min. At 41–44 °C, burst drug release was observed with a maximum of 64% of encapsulated drug released after 270 s at 42 °C.

### Sensitivity of NSCLC cells to HT and LUM

To determine the sensitivity of NSCLC H460 and H520 cells to HT, cells were incubated at 37 °C or exposed to 42.0 ± 0.6 °C for 1 h (followed by incubation at 37 °C) (Table 2). HT had a significant effect on H460 cell viability, resulting in a 21.6 ± 4.1% decrease in cell viability (p = 0.002). In contrast, the effect of HT in H520 cells was not statistically significant, with a decrease in cell viability of only 6.8 ± 8.1% (p = 0.3).

**Table 2:** Cell viability in the presence and absence of LUM, HT, CDDP, and VRL.

	H460	H520
HT induced cell death	21.6 ± 4.1%*	6.8 ± 8.1%
LUM IC50 (nM)	11.5 ± 3.1	12.8 ± 2.1
LUM + HT IC50 (nM)	9.2 ± 2.3	12.3 ± 1.5
CDDP IC50 (µM)	1.3 ± 0.3	6.5 ± 0.8
VRL IC50 (nM)	8.3 ± 2.0	3.4 ± 1.4

Error represents the SD between at least three independent experiments (*p < 0.01 relative to untreated control).

The cells were also exposed to LUM ± HT to determine HT’s effect on the cytotoxicity of LUM. In the absence of HT, LUM was found to have IC_50_ values in the low nanomolar range in both H460 and H520 cells. A 1 h exposure to HT in addition to a 72 h exposure to LUM resulted in an insignificant reduction in IC_50_ of LUM in both H460 (p = 0.3) and H520 (p = 0.8) cells.

### Sensitivity of NSCLC cells to combinations of LUM + CDDP and LUM + VRL

**Figure 4:**
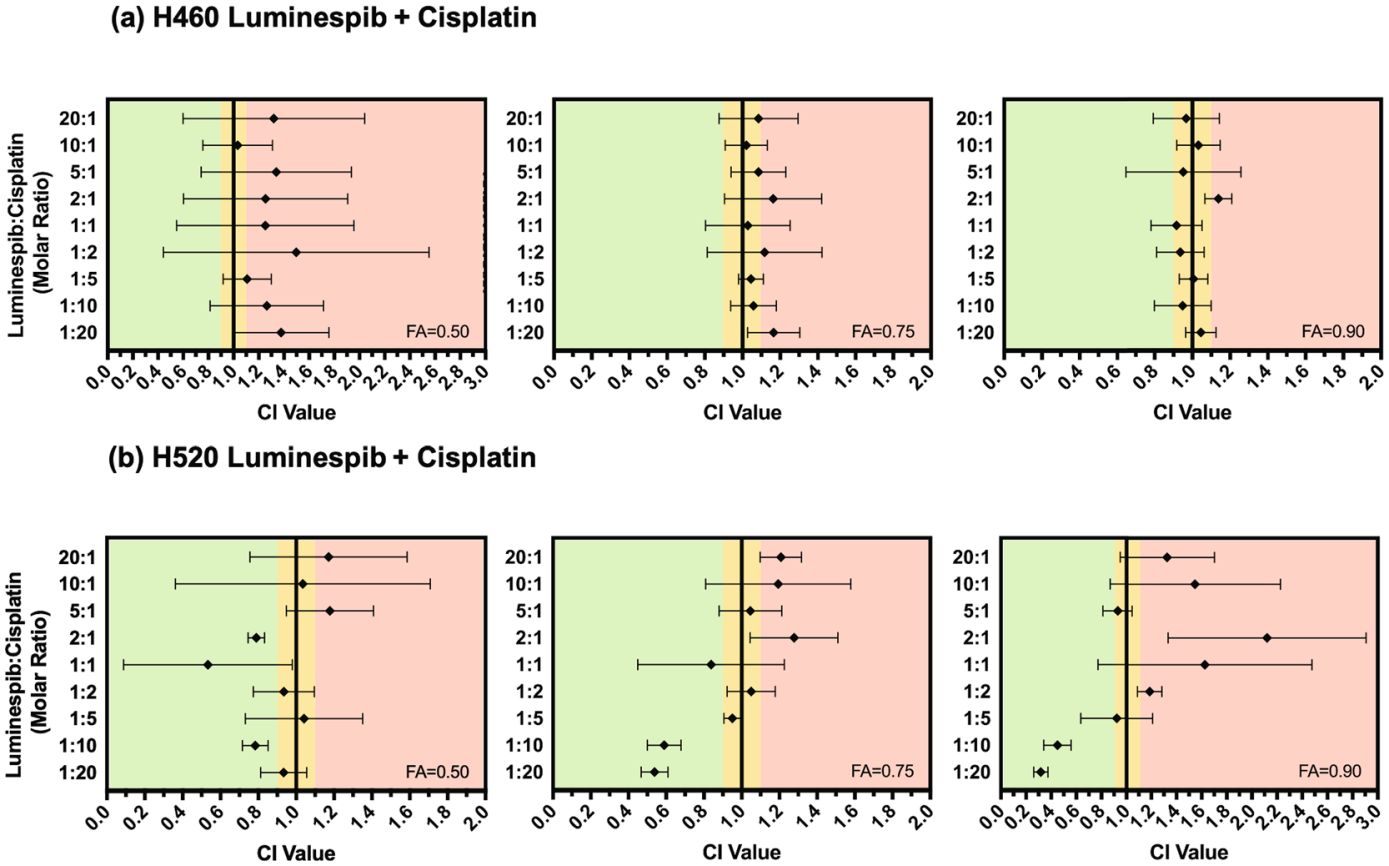
CI values for H460 (**a**) and H520 (**b**) cells treated with various molar ratios of LUM + CDDP at fraction affected (*FA*) = 0.50, 0.75, and 0.90. Data are presented as mean and SD (n = 3). CI values < 0.90 indicate that the two drugs act synergistically (shown in green) while CI values of 0.90–1.10 indicate an additive effect (shown in yellow), and CI values > 1.10 indicate that the two drugs act antagonistically (shown in red). The combination was found to be mostly additive and antagonistic in both cell lines, except 1:10 and 1:20 LUM:CDDP, which were found to have a synergistic effect in H520 cells.

**Figure 5:**
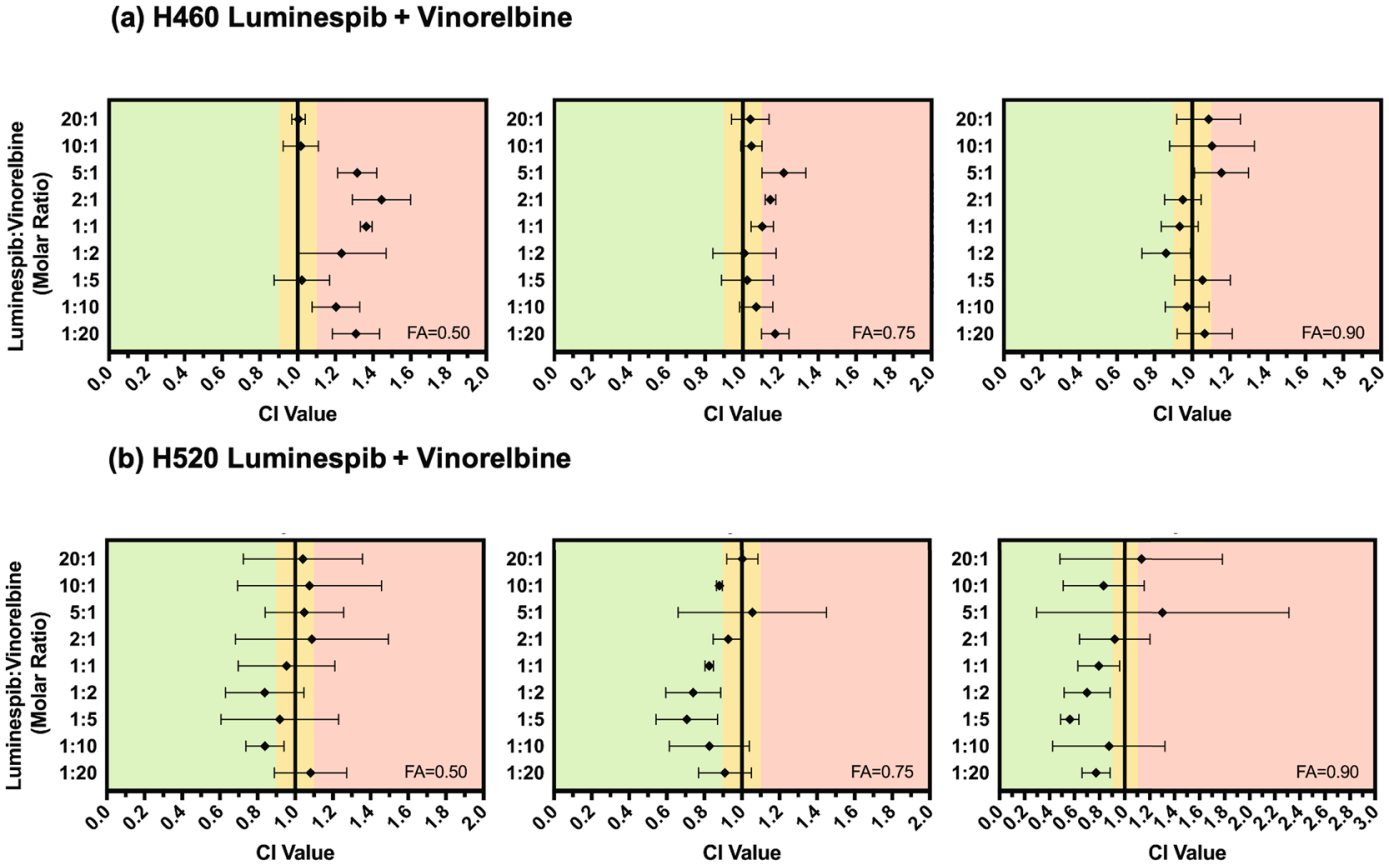
CI values for H460 (**a**) and H520 (**b**) cells treated with various molar ratios of LUM + VRL at *FA* = 0.50, 0.75, and 0.90. Data are presented as mean and SD (n ≥ 3). CI values < 0.90 indicate that the two drugs act synergistically (shown in green) while CI values of 0.90–1.10 indicate an additive effect (shown in yellow), and CI values > 1.10 indicate that the two drugs act antagonistically (shown in red). The combination was found to be additive and antagonistic in H460 cells, while most ratios were additive and synergistic in H520 cells.

To determine the effect of combination treatments, the IC_50_ of both CDDP and VRL monotherapies was first determined. As shown in Table 2, the IC_50_ of CDDP was found to be in the low micromolar range in both H460 and H520 cells, whereas the IC_50_ of VRL was found to be in the low nanomolar range in both cell lines. In order to determine any potential synergistic activity, the sensitivity of the two cell lines to a combination of LUM + CDDP (Fig. 4), as well as LUM + VRL (Fig. 5), was assessed. In H460, LUM + CDDP was found to have an additive or antagonistic effect across all molar ratios of drug (1:20 to 20:1 LUM:CDDP). In H520, LUM + CDDP was found to be additive or antagonistic at most ratios. Interestingly, molar ratios of 1:10 and 1:20 LUM:CDDP were found to result in a synergistic effect. In H460, LUM + VRL was found to be additive or antagonistic while in H520, the combination was generally observed to be additive or synergistic for most molar ratios, except 20:1 and 5:1 LUM:VRL, which resulted in more antagonistic CI values.

## Discussion

LUM is one of the most potent HSP90i and is highly cytotoxic in vitro and in vivo in various cancer cells both as a monotherapy or in combination with chemotherapy, molecular therapies, and radiation^13,18,20,63–70^. Despite promising preclinical potency, clinical trials revealed insufficient efficacy and a high occurrence of ocular toxicities^21,23–32^. Novel formulation strategies are required in order to improve the therapeutic index for this drug. For the first time, LUM has been encapsulated in a thermosensitive liposome to provide localized drug delivery and allow LUM to reach its full clinical potential. HT-triggered drug release is generally accomplished in vivo using temperatures in the range of 39-45 °C^55^, as explored in this paper. Employing this strategy, thermosensitive liposomes have been shown to improve drug delivery efficiency compared to either free or “traditional” liposome-encapsulated drug in combination with HT^71–73^. Drug retention issues observed with traditional liposomes are overcome with this heat-triggered drug release. As discussed by Drummond et al., the encapsulated drug is considered to be in the “inactive”, or “prodrug” form, thus unable to elicit an effect^74^. HT is utilized to release encapsulated drugs, which can then reach the therapeutic target. Single chained lysolipids are included in many formulations to aid in rapid drug release, which is desirable given that the liposomes must release their contents as they pass through the tumor vasculature in a matter of seconds^75^. In this study, the lysolipid-containing lipid composition of ThermoDox was used to prepare thermoLUM. This lipid composition has been used in various clinical trials evaluating ThermoDox and would facilitate the clinical translation of a thermosensitive formulation containing LUM. The in vitro release studies demonstrated the rapid release of LUM upon heating the liposomes to temperatures in the range of HT, while the formulation remained stable (i.e., < 15% release) at body temperature. ThermoLUM was also found to be stable over a 3-week period under different storage conditions. The robust storage stability of this formulation will facilitate upcoming preclinical studies as well as enabling the possibility for future clinical translation.

LUM is the second HSP90i to be encapsulated inside a thermosensitive liposome, the first being alvespimycin^36^. Tanespimycin has also been formulated in a thermosensitive liposome however, the hydrophobic molecule was incorporated into the lipid bilayer^41^. ThermoLUM liposomes were found to have a physicochemical profile comparable to other thermosensitive liposome formulations, in terms of size, ζ-potential, and lipid bilayer transition temperature^36,46,49^. A high drug-to-lipid ratio (1:24) was achieved through exploitation of the ionizable nature of LUM, which allowed the molecule to be actively loaded. LUM was encapsulated at a similar drug-to-lipid ratio to thermosensitive liposome formulations encapsulating other molecules^36,46,48,76^. Heat triggered release was found to begin at 40 °C and maximized by 42 °C with over 50% of encapsulated drug released in the first 30 s and just over 60% of drug released within 5 min. This release profile differs from other formulations prepared with the ThermoDox lipid composition, where the burst release is closer to 100%^36,46–48,77^. Although the liposomes in this study demonstrated incomplete release (an issue inherently associated with low efficacy in traditional liposomes such as Doxil^43^), thermoLUM still demonstrated a rapid burst-release profile once heated at mild HT temperatures. Nonetheless, this should result in therapeutic levels of LUM at the tumor site because LUM is substantially more cytotoxic to NSCLC cells compared to other common chemotherapeutics encapsulated into thermosensitive liposome formulations, such as doxorubicin^78^ and CDDP, that have IC_50_ values in the high nanomolar and low micromolar range. Therefore, at a similar drug to lipid ratio, an incomplete release would still result in an efficacious level of LUM delivered to the tumor, whereas this extent of release would pose an issue with its chemotherapeutic counterparts. The efficacy and toxicity of thermoLUM will need to be evaluated in vivo to demonstrate the advantages associated with delivering LUM in a thermosensitive nanoparticle, however, these results are promising. This drug-to-lipid ratio provides drug levels feasible for future in vivo efficacy and toxicity studies. At a lipid concentration of 60 mg/mL, as our group has used previously^79^, the LUM concentration would be 1.45 mg/mL and easily allow a 14.5 mg/kg dose (200 µL, 0.29 mg LUM) to be administered to mice. Our previous results found thermosensitive liposomes delivering 3.3% of the injected dose to 150 mg tumors^36^. Accounting for our 64% drug release efficiency and assuming uniform drug distribution within the tumor, this treatment strategy is predicted to produce a LUM concentration of 87 µM in the tumor (equivalent to 87 nmol/g tumor), considerably exceeding the IC_50_ of LUM in either H520 or H460 cell lines. A previous preclinical study of LUM in mice bearing breast cancer tumors resulted in a maximum tumor concentration of 16.36 nmol/g tumor at an intravenous dose of 30 mg/kg free LUM^18^, which is comparable to the human dose of 70 mg/m^2^^23^. Therefore, at less than half of the dose, thermoLUM has the potential to deliver more than 5 times the amount of drug to the tumor.

While HT is utilized to trigger release from thermosensitive liposomes, there are many other benefits of using HT as a treatment modality. HT has been proven to enhance both radiation and chemotherapeutic treatments by improving blood flow and tumor microvasculature permeability^55^. HT also has direct cytotoxic effects as a monotherapy which is dependent on exposure time and temperature^71,72,80^. In the current study, a 1 h exposure to 42 °C was found to have a cytotoxic effect on the H460 cells, but no significant effect was observed in the H520 cells. It has previously been found that colorectal cancer cells with mutant KRAS are more sensitive to HT (exposure to 42 °C for 24 h) than cells with wild-type KRAS^80^. H460 cells are found to be KRAS mutant, while H520 cells are KRAS wild-type. This difference may offer a possible explanation for the differential sensitivity to HT as a monotherapy. HT has also been shown to increase the cytotoxicity of common chemotherapeutics and molecular therapeutics in vitro in a cell-line dependent manner^36,71,72^. While brief exposure to HT did not increase the cytotoxicity of LUM in vitro, the addition of HT may lead to a significant increase in tumor drug delivery.

Thermosensitive drug delivery has seldom been explored for the treatment of NSCLC because lung lesions have historically been considered very challenging to treat with HT. Previously, obstacles such as respiratory movement and ultrasound interference in the air-filled lung cavities have limited the use of HT. However, current research is making thermal therapies such as microwave, radiofrequency, and focused ultrasound viable options for heating lung tissue^81–85^. Indeed, a clinical trial combining focused ultrasound-induced HT and PD-1 antibody blockade is now recruiting patients with small cell and non-small cell lung cancer, among other solid tumors (NCT04116320). ThermoDox has demonstrated efficacy independent of the applied heating technique. Therefore, it is plausible that NSCLC patients who are eligible to undergo microwave, radiofrequency, or focused ultrasound-induced HT treatment, may also receive thermosensitive liposomes containing LUM in a manner similar to patients receiving ThermoDox.

When administered as free drug, LUM displayed systemic toxicity and a lack of efficacy which led to the discontinuation of clinical development. By improving the distribution of LUM through delivery via thermosensitive liposomes, new therapeutic strategies involving this potent HSP90i are possible. One of these strategies is to combine LUM with existing chemotherapeutic agents currently employed in the clinic. Given that both VRL and CDDP are used in the treatment of NSCLC, in vitro activity of each agent in combination with LUM was assessed. Our group has previously formulated both VRL and CDDP thermosensitive liposomes^46,79^. Therefore, it would be feasible to administer thermoLUM with either of these formulations, in order to obtain a synergistic or additive effect. In vitro, H460 and H520 cells displayed similar sensitivity to LUM, VRL, and CDDP monotherapies as previously reported^63,78,79,86,87^.

The combination of LUM + VRL was mostly additive and antagonistic in H460 cells, while mostly additive and synergistic in H520 cells (Fig. 5). LUM and VRL have not been combined previously; however, HSP90i have previously been studied in combination with other tubulin inhibitors. The HSP90i ganetespib showed promising preclinical synergy with tubulin inhibitors paclitaxel, docetaxel, and vincristine in NSCLC cells^52^. In a Phase II clinical trial (NCT01348126), ganetespib was combined with docetaxel in patients with advanced NSCLC and resulted in improvements in both progression-free survival (PFS) as well as overall survival (OS)^88^. This led to the Phase III clinical trial (NCT01798485) in NSCLC patients; however no significant improvements in OS and PFS were observed in this study^89^.

Although most drug ratios in this study were found to be antagonistic or additive, LUM + CDDP was synergistic at both 1:10 and 1:20 ratio of LUM:CDDP in H520 cells (Fig. 4). LUM has been studied in combination with CDDP previously. The combination of LUM + CDDP was found to be weakly additive in adrenocortical carcinoma cells^90^. A study combining LUM with CDDP + gemcitabine in a breast cancer in vivo PDX model, found the triple combination resulted in a complete response, whereas LUM as a monotherapy and CDDP + gemcitabine as a dual-therapy did not halt disease progression^67^. However, no further studies have been pursued with this combination. In other studies, LUM was found to significantly sensitize head and neck cancer cells to CDDP, radiation, and a combination of CDDP + radiation^68^, and esophageal adenocarcinoma cells to a combination of CDDP + 5-fluorouracil^13^. Preclinical studies with other HSP90i have also demonstrated cell line dependent effects of combinations with CDDP. When HSP90i tanespimycin was combined with CDDP in a panel of colon adenocarcinoma cell lines, synergistic and additive effects were found in some cell lines. In contrast, antagonistic effects were seen in others, which appeared to be dependent on the extent of inhibition of apoptotic signaling by tanespimycin^91^.

The current study demonstrates that LUM is synergistic with VRL and CDDP when administered at specific molar ratios. Both combinations were found to result in greater synergy in H520 cells and more antagonism in H460 cells. These differences highlight the importance of carefully selecting drug combinations for the right patient population, to avoid administering an antagonistic combination. Vyxeos^®^ is the first FDA-approved liposomal formulation to encapsulate two anti-cancer agents, cytarabine and daunorubicin, at a specific molar ratio (5:1 cytarabine:daunorubicin). Despite the combination resulting in antagonism in some cell lines, the specific ratio that was synergistic in the majority of cell lines was chosen for further studies. The formulation that encapsulated that specific ratio was approved for the treatment of adults with acute myeloid leukemia^92^. Although some promising molar ratios of combinations were found in this study, these two drug combinations need to be studied in a much larger panel of cell lines before moving to a preclinical in vivo model with a preferred molecular signature. Further studies are needed to investigate the underlying mechanisms which led to the differential drug combination effects observed in the two cell lines.

In conclusion, LUM was successfully encapsulated into thermosensitive liposomes that provided quick and efficient heat-activated drug release in response to standard HT temperatures. In vitro studies determined that careful selection of drug ratios resulted in synergistic activity when combining LUM with CDDP or VRL, which are standard of care chemotherapeutics for treating NSCLC. As our group has previously formulated CDDP and VRL in thermosensitive liposomes, it is our belief that this formulation strategy will serve to improve the anticancer potential of LUM.

## Materials and methods

### Materials

MSPC, DPPC, and mPEG_2000_-DSPE were purchased from CordenPharma Switzerland (Liestal, CH). LUM and VRL were purchased from Selleck Chemicals (Houston, TX). Sucrose octasulphate (sodium salt) was purchased from Toronto Research Chemicals (North York, ON). BSA (heat shock fraction, pH 7, ≥ 98%), CDDP, Dowex 50WX8-200 resin, fetal bovine serum (FBS), methanesulfonic acid, penicillin and streptomycin (P/S), phenazine ethosulfate (PES), RPMI-1640 medium (with l-glutamine and sodium bicarbonate), triethylamine (TEA), and were purchased from Sigma-Aldrich (Oakville, ON). Sepharose CL-4B agarose size exclusion chromatography base matrix was purchased from GE Healthcare Bio-Sciences (Uppsala, SE). NCL-H460 and NCL-H520 NSCLC cells were purchased from ATCC (Manassas, VA). CellTiter 96^®^ AQueous 3-(4,5-dimethylthiazol-2-yl)-5-(3-carboxymethoxyphenyl)-2-(4-sulfophenyl)-2H-tetrazolium (MTS) Reagent Powder was purchased from Promega (Madison, WI).

### HPLC analysis

High-performance liquid chromatography (HPLC) was used to quantify LUM. The system consisted of an Agilent Technologies 1260 Infinity II HPLC with a diode array detector (DAD), Agilent Eclipse XDB-C18 analytical guard column (4.6 × 12.5 mm, 5 μm), and an Agilent Eclipse XDB-C18 column (4.6 × 150 mm, 5 μm) (Agilent, Mississauga, ON). The mobile phase consisted of an aqueous phase (0.1% formic acid in deionized water) and an organic phase (0.1% formic acid in acetonitrile) in a ratio of 70:30. Isocratic elution at a flow rate of 1 mL/min, and a detection wavelength of 310 nm were used to detect the drug.

### Luminespib salt preparation

LUM was dissolved in methanol (MeOH) at 2 mg/mL. Methanesulfonic acid (MsOH) was added at a 1:1.2 molar ratio of LUM to MsOH in MeOH. The solution was vortexed vigorously and left at RT for 24 h to allow the MeOH to evaporate. Water was added to obtain a concentration of 5 mg/mL LUM. To remove water-insoluble LUM, the solution was centrifuged at 14,000 rpm for 5 min. The supernatant was analyzed via HPLC to determine the final concentration of LUM. The mesylate salt of LUM was used for all experiments.

### TEA_8_SOS preparation

Triethylamine sucrose octasulphate (TEA_8_SOS) was prepared as previously described^74^. In brief, sodium octasulphate was added to Dowex 50WX8-200 resin. Neat TEA was then used to titrate the eluted free acid. The resulting TEA_8_SOS was diluted with deionized water to a final sulphate group concentration of 0.65 M.

### Liposome preparation

Thermosensitive liposomes were prepared as previously described^36,74,93^. Briefly, DPPC, MSPC, and mPEG_2000_-DSPE were dissolved at an 86:10:4 molar ratio in chloroform. The chloroform was evaporated under nitrogen gas, and the resulting lipid film was further dried under vacuum overnight. The film was then hydrated in 0.65 M TEA_8_SOS (pH 5.7) at 60 °C for 1 h resulting in a lipid concentration of 0.125 M. The liposomes were extruded at 55 °C using a 10 mL Lipex extruder (Northern Lipids Inc., Vancouver, BC) 3 times through two stacked 200 nm pore size track-etch polycarbonate membranes (Whatman Inc., Clifton, NJ) at 200 psi, and 10 times through two stacked 100 nm membranes at 400 psi. The unloaded liposomes were dialyzed at 4 °C overnight against a 500-fold volume excess of HBS solution (150 mM sodium chloride, 20 mM HEPES, pH 7.4) using 50 kDa molecular weight cut-off dialysis tubing in order to exchange the external buffer.

### Drug loading

LUM mesylate was actively loaded into thermosensitive liposomes. The liposomes were pre-heated at 35 °C for 10 min. LUM was then added at a 1:20 drug-to-lipid molar ratio and incubated at 35 °C for 1 h. The liposomes were dialyzed at 4 °C overnight against a 500-fold volume excess of HBS (pH 7.4) using 50 kDa molecular weight cut-off dialysis tubing to exchange the external buffer and remove unencapsulated drug.

### Drug molecules per liposome calculation

The approximate number of drug molecules per liposome was calculated as previously described^94^ using the following equations:

1 $${A}_{weighted}=\frac{{A}_{DPPC}\left({mol\%}_{DPPC}\right)+{A}_{MSPC}\left({mol\%}_{MSPC}\right) {+ A}_{mPEG-DSPE}\left({mol\%}_{mPEG-DSPE}\right)}{100\%}.$$

2 $${l}_{om}=\frac{4\pi {{r}_{v}}^{2}}{{A}_{weighted}}$$

3 $${l}_{im}=\frac{4\pi {({r}_{v}-{d}_{b})}^{2}}{{A}_{weighted}}$$

4 $${Q}_{LUM}=\left({l}_{om}+{l}_{im}\right)\left(\frac{D}{L}\right)$$

where *A*_*weighted*_ is the weighted average area of the membrane occupied by a single lipid; *A*_*DPPC*_, *A*_*MSPC*_*, A*_*mPEG-DSPE*_ represent the area per membrane phospholipid molecule and were estimated to be 4.94 × 10^–19^^95^, 4.80 × 10^–19^^96^ and 5.00 × 10^–19^ m^2^^97^, respectively; *A*_*weighted*_ was calculated to be 4.93 × 10^–19^ m^2^; *l*_*om*_ is the number of lipids in the outer membrane of the lipid bilayer; *r*_*v*_ represents the vesicle radius, in meters, determined by dynamic light scattering (DLS); *l*_*im*_ is the number of lipids in the inner membrane; *d*_*b*_ is the bilayer thickness which is estimated to be 3.93 × 10^–19^ m ^98^; *Q*_*LUM*_ is the quantity of LUM molecules per vesicle, and; D/L is the drug to lipid molar ratio.

### Size and zeta (ζ)-potential

The size and PDI of the liposomes at a 100-fold dilution in phosphate-buffered saline (PBS) were determined by DLS (Zeta Sizer Nano-ZS, Malvern Instruments Ltd., Malvern, UK). This instrument was also used to determine the ζ-potential of the liposomes at a 100-fold dilution in deionized water.

### Transition temperature

The T_m_ of the lipid bilayer was determined using a Q100 TA dynamic scanning calorimeter (DSC) (TA Instruments, New Castle, DE) by heating the sample at a rate of 1 °C/min from 25 to 60 °C.

### Cryogenic transmission electron microscopy (cryo-TEM)

Samples were prepared by pipetting 5 μL of liposomes on a Quantifoil Multi A holey carbon film supported by a copper grid (Quantifoil Micro Tools GmbH, Jena, Germany). The samples were immediately frozen by immersion into liquid ethane, then transferred under liquid nitrogen to a FEI Tecnai G2 F20 microscope (FEI Company, Hillsboro, OR). Images were captured at approximately − 170 °C with a Gatan CCD camera (Gatan Inc., Warrendale, PA) and a 200 kV acceleration voltage.

### Long-term stability

To assess the stability of the thermosensitive liposomes, thermoLUM was stored at RT (approximately 22 °C and 4 °C for 21 days. The size, PDI, and amount of encapsulated drug were determined on the day of preparation, and after 1, 4, 6, 7, 14 and 21 days of storage at either RT or 4 °C. Liposome size and PDI were determined by DLS as described above. The amount of encapsulated LUM was determined by passing aliquots of liposomes through size exclusion chromatography columns (Sepharose CL-4B agarose) to separate the encapsulated drug from unencapsulated drug. The concentration of encapsulated LUM was determined by HPLC analysis, as described above.

### In vitro drug release

To determine the stability of the thermosensitive liposomes at physiologically relevant temperatures, as well as the ability of the liposomes to release the encapsulated drug at HT temperatures, a release study was conducted in 45 mg/mL BSA in PBS. Liposomes were added at a 20-fold dilution to BSA pre-heated to 37–44 °C. At 30 s intervals, aliquots from the release media were passed through size exclusion chromatography columns (Sepharose CL-4B agarose) to separate the encapsulated drug from unencapsulated drug. Samples from the size exclusion column were lyophilized overnight (Freezone 4.5, Labconco, Kansas City, Missouri). The dried samples were then rehydrated in MeOH, vortexed, and centrifuged at 14,000 rpm for 30 min. Following this, the resulting supernatant was analyzed via HPLC. The resulting data were fitted on GraphPad Prism version 7.0 with the following first-order equation:

5 $$R\left(t\right)={R}_{max}(1-{e}^{-kt})$$

where *R*(*t*) represents the percentage of the drug (i.e., LUM) released per unit of time (*t*); *R*_*max*_ represents maximum drug (i.e., LUM) released; and the release rate constant is denoted by *k*.

### In vitro cytotoxicity

H460 and H520 NSCLC cells were cultured in RPMI medium supplemented with 1% P/S and 10% FBS, at 37 °C and 5% CO_2_ unless otherwise specified. All cell lines were authenticated using STR profiling by the Centre for Applied Genomics Genetic Analysis Facility (TCAG, Toronto). The MTS assay was used to determine cell viability^99^. Briefly, H460 and H520 cells were seeded in 96-well plates and incubated overnight at densities of 1000 and 5000 cells/well, respectively. Cells were treated with either LUM, VRL, CDDP, or a combination of two drugs for 72 h. A 2 mg/mL MTS solution containing 0.21 mg/mL PES was added to the cells and incubated for 1 h. A Cytation 5 Cell Imaging Multi-Mode Reader (BioTek, Winooski, VT) was used to measure UV absorbance at 490 nm. Individual data points were normalized to positive and negative controls. To determine IC_50_ values, the resulting data were fitted to a dose–response curve (4-parameter sigmoidal) in GraphPad Prism (V. 7.0).

The method developed by Chou and Talalay^58,100^, was used to determine the effect of combining either LUM and VRL or LUM and CDDP at various molar ratios in H460 and H520 cells. The combination indices (CI) for various fractions of affected cells (*FA*) were calculated computationally using CompuSyn software (ComboSyn Inc., Paramus, NJ) with the following equation:

6 $${CI}_{FA}= \frac{{\left({IC}_{50}\right)}_{LD}{\left(\frac{FA}{1-FA}\right)}^{1/{m}_{LD}}\left(\frac{L}{L+D}\right)}{{\left({IC}_{50}\right)}_{L}{\left(\frac{FA}{1-FA}\right)}^{1/{m}_{L}}}+\frac{{\left({IC}_{50}\right)}_{LD}{\left(\frac{FA}{1-FA}\right)}^{1/{m}_{LD}}\left(\frac{D}{L+D}\right)}{{\left({IC}_{50}\right)}_{D}{\left(\frac{FA}{1-FA}\right)}^{1/{m}_{D}}}$$

where (*IC*_*50*_) represents the concentration of the drug or of the drug combination required to produce 50% cell inhibition; the subscript *LD* refers to the combination of L (LUM) and D (other drugs, i.e., CDDP or VRL); *FA* represents the fraction of cells affected by the drug treatment; *m* represents the slope of the median effect plot for the drug or of the drug combination (i.e., where x = log(dose) and y = log(*FA*/1 − *FA*)); L/L + D represents the portion of the total drug treatment that is LUM, and; D/L + D represents the portion of the total drug treatment that is the other drug. CI values < 0.90 indicate that the two drugs act synergistically at that ratio and *FA*, while CI values of 0.90–1.10 indicate an additive effect, and CI values > 1.10 indicate that the two drugs act antagonistically at that ratio and *FA*.

### In vitro HT

To determine the effect of HT, cells were incubated, in the presence and absence of LUM, at 42.0 ± 0.6  °C for 1 h, followed by 37  °C for the remainder of the 72 h. The temperature was monitored in a 96-well plate containing an equivalent volume of media using an external temperature probe (Traceable Kangaroo™ Thermometer, Thomas Scientific, Swedesboro, NJ).

### Statistical analysis

Statistical analysis was performed using GraphPad Prism version 7.0. The t test (two-tailed, unpaired) was used to calculate the statistical significance of differences between IC_50_ values of LUM and LUM + HT in H460 and H520 cell monolayers. Values were considered significantly different when p < 0.05.

## Supplementary Information

Supplementary Information 1.

## Data Availability

The datasets generated and analyzed during the current study can be made available upon request.
