# Supplementary material for: Heat-activated nanomedicine formulation improves the anticancer potential of the HSP90 inhibitor luminespib in vitro
Source: Sci Rep. 2021 May 27;11:11103. doi: 10.1038/s41598-021-90585-w (PMC8160139; doi:10.1038/s41598-021-90585-w)
Supplement: Supplementary file 1 — Supplementary Information 1. [file 41598_2021_90585_MOESM1_ESM.pdf]

# Heat-activated nanomedicine formulation improves the anticancer potential of the HSP90 inhibitor luminespib *in vitro*

Brittany Epp-Ducharme[1], Michael Dunne[1], Linyu Fan[1], James C. Evans[1], Lubabah Ahmed[1], Pauric Bannigan[1], Christine Allen[1*]

[1] Leslie Dan Faculty of Pharmacy, University of Toronto, Toronto, ON M5S 3M2, Canada

## Supplementary Information

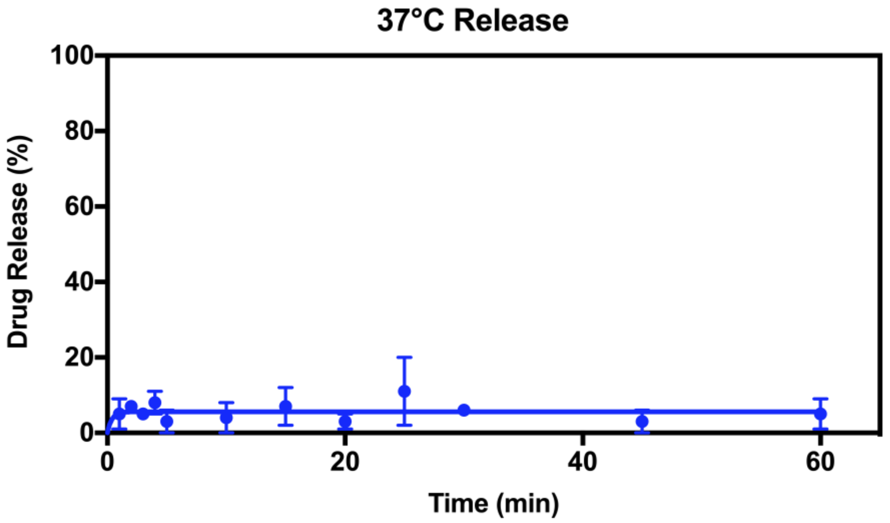


**Supplementary Figure S1:** Drug release from thermoLUM incubated at 37°C in 45 mg/mL BSA over 60 min. Samples were collected at 30 s intervals for 5 min, followed by 5 min intervals until 30 min, and 15 min intervals until 60 min. The liposome-encapsulated drug was separated from unencapsulated drug via size exclusion chromatography. The concentration of LUM was detected via HPLC analysis to determine the amount of drug release from the liposomes at each timepoint. The liposomes were found to release less than 15% of drug over 1 h. Data shown represent the mean ± SD (n=3).

**Supplementary Table S1:** CI values for H460 and H520 cells treated with various molar ratios of LUM + VRL and LUM + CDDP at FA=0.50, 0.75, and 0.90. Data are presented as mean and SD (n≥3). CI values <0.90 indicate that the two drugs act synergistically while CI values of 0.90-1.10 indicate an additive effect, and CI values >1.10 indicate that the two drugs act antagonistically.

| | | **CI Value** | | | | | |
|---|---|---|---|---|---|---|---|
| | **Drug Ratio** | **FA=0.50** | | **FA=0.75** | | **FA=0.90** | |
| **Cell Line** | **LUM:VRL** | **Mean** | **SD** | **Mean** | **SD** | **Mean** | **SD** |
| H460 | 1:1 | 1.36 | 0.03 | 1.10 | 0.06 | 0.93 | 0.10 |
| | 1:2 | 1.23 | 0.24 | 1.01 | 0.17 | 0.86 | 0.13 |
| | 2:1 | 1.45 | 0.15 | 1.15 | 0.03 | 0.95 | 0.10 |
| | 1:5 | 1.02 | 0.15 | 1.02 | 0.14 | 1.05 | 0.15 |
| | 5:1 | 1.32 | 0.10 | 1.22 | 0.12 | 1.16 | 0.14 |
| | 1:10 | 1.20 | 0.13 | 1.07 | 0.09 | 0.97 | 0.12 |
| | 10:1 | 1.02 | 0.09 | 1.05 | 0.06 | 1.11 | 0.22 |
| | 1:20 | 1.31 | 0.12 | 1.17 | 0.07 | 1.07 | 0.15 |
| | 20:1 | 1.00 | 0.04 | 1.04 | 0.10 | 1.09 | 0.17 |
| H520 | 1:1 | 0.95 | 0.26 | 0.83 | 0.02 | 0.79 | 0.17 |
| | 1:2 | 0.84 | 0.21 | 0.74 | 0.15 | 0.70 | 0.18 |
| | 2:1 | 1.09 | 0.41 | 0.93 | 0.08 | 0.92 | 0.28 |
| | 1:5 | 0.92 | 0.31 | 0.71 | 0.16 | 0.56 | 0.07 |
| | 5:1 | 1.05 | 0.21 | 1.06 | 0.40 | 1.30 | 1.01 |
| | 1:10 | 0.84 | 0.10 | 0.83 | 0.21 | 0.88 | 0.45 |
| | 10:1 | 1.08 | 0.38 | 0.88 | 0.02 | 0.83 | 0.32 |
| | 1:20 | 1.08 | 0.19 | 0.91 | 0.14 | 0.77 | 0.11 |
| | 20:1 | 1.04 | 0.32 | 1.00 | 0.08 | 1.13 | 0.65 |
| | **LUM:CDDP** | | | | | | |
| H460 | 1:1 | 1.25 | 0.70 | 1.03 | 0.22 | 0.92 | 0.14 |
| | 1:2 | 1.50 | 1.05 | 1.12 | 0.30 | 0.94 | 0.13 |
| | 2:1 | 1.25 | 0.65 | 1.16 | 0.26 | 1.14 | 0.07 |
| | 1:5 | 1.11 | 0.19 | 1.05 | 0.07 | 1.01 | 0.08 |
| | 5:1 | 1.34 | 0.60 | 1.08 | 0.15 | 0.95 | 0.31 |
| | 1:10 | 1.26 | 0.45 | 1.06 | 0.12 | 0.95 | 0.15 |
| | 10:1 | 1.03 | 0.28 | 1.02 | 0.11 | 1.03 | 0.12 |
| | 1:20 | 1.38 | 0.38 | 1.17 | 0.14 | 1.05 | 0.08 |
| | 20:1 | 1.32 | 0.72 | 1.09 | 0.21 | 0.97 | 0.18 |
| H520 | 1:1 | 0.53 | 0.45 | 0.84 | 0.39 | 1.63 | 0.85 |
| | 1:2 | 0.93 | 0.16 | 1.05 | 0.13 | 1.19 | 0.10 |
| | 2:1 | 0.79 | 0.04 | 1.28 | 0.23 | 2.12 | 0.79 |

| | | | | | | | | | |
|---|---|---|---|---|---|---|---|---|---|
| | 1:5 | 1.04 | 0.31 | | 0.95 | 0.04 | | 0.92 | 0.29 |
| | 5:1 | 1.18 | 0.23 | | 1.05 | 0.17 | | 0.93 | 0.12 |
| | 1:10 | 0.78 | 0.07 | | 0.59 | 0.09 | | 0.45 | 0.11 |
| | 10:1 | 1.04 | 0.67 | | 1.19 | 0.38 | | 1.55 | 0.68 |
| | 1:20 | 0.93 | 0.12 | | 0.54 | 0.07 | | 0.32 | 0.06 |
| | 20:1 | 1.17 | 0.41 | | 1.21 | 0.11 | | 1.33 | 0.38 |
